# Two-Item Sentence Comprehension by a Dog (*Canis familiaris*)

**DOI:** 10.1371/journal.pone.0029689

**Published:** 2012-02-01

**Authors:** Daniela Ramos, Cesar Ades

**Affiliations:** 1 Department of Medical Clinics, Faculty of Veterinary Medicine and Animal Sciences, University of São Paulo, São Paulo, São Paulo, Brazil; 2 Department of Experimental Psychology, Institute of Psychology, University of São Paulo, São Paulo, São Paulo, Brazil; University of Arizona, United States of America

## Abstract

Syntax use by non-human animals remains a controversial issue. We present here evidence that a dog may respond to verbal requests composed of two independent terms, one referring to an object and the other to an action to be performed relative to the object. A female mongrel dog, Sofia, was initially trained to respond to action (*point* and *fetch*) and object (*ball*, *key*, *stick*, *bottle* and *bear*) terms which were then presented as simultaneous, combinatorial requests (e.g. *ball fetch*, *stick point*). Sofia successfully responded to object-action requests presented as single sentences, and was able to flexibly generalize her performance across different contexts. These results provide empirical evidence that dogs are able to extract the information contained in complex messages and to integrate it in directed performance, an ability which is shared with other linguistically trained animals and may represent a forerunner of syntactic functioning.

## Introduction

Dogs are endowed with a special ability to understand human signals. A large amount of research has shown that they can, very early in ontogeny, successfully use social cues such as pointing or gazing or body posture to locate hidden food [1], [2], [3]. Human language constitutes another very common source of signals to which dogs appear to be remarkably responsive, but which is still insufficiently investigated. Owners report that their dogs have a very developed understanding of words [4], [5] and verbal commands are widely and successfully used by dog trainers to induce and control a variety of behaviors. We know that word discrimination may be affected by the human sender's attentional status and appearance [6], [7], by nonverbal signals that go along with words [7] and by small changes in the phoneme composition of words [8]. Early [9], [10], [11] and more recent investigations [12] examined dogs' capacity to discriminate words associated with different objects, places and performances, with an interest in referential knowledge. A high level of competence in word comprehension has been demonstrated in some dogs: Rico, a border collie, was able to discriminate dozens of words and showed a “fast-mapping” performance similar to children's, that is, the capacity of attributing, by exclusion, a new word to an object never seen before [12]. A critical evaluation of an interpretation in terms of fast mapping has been proposed, though [13]. Betsy, another family trained border collie, fetched any object in a set of hundreds when prompted through verbal labels, and could use photographs as sufficient information about items to be searched for [14]; more recently, Chaser, trained over a period of three years, was able to learn and retain the names of more than a thousand objects and to use words representing object categories [15].

Dogs' responsiveness to words has been examined either under [1] *action request* conditions (dogs are required to perform an action when prompted by a verbal command: “sit”, “roll”, “give paw”, “fetch”); [2] *object request* conditions (they are trained to react selectively to one of several objects according to a verbal command such as “fetch the ball, the teddy, the newspaper, etc.”) [12], [14]. In both cases, requests are composed either of single words or short phrases which may be functionally equivalent to single words [6], [8], [12], [16].

This single-item/single-response acquisition indicates the existence of a word-object or word-action mapping process but falls short of showing one of the most distinctive features of human verbal communication, the fact that it is composed of *sentences*, built up in a combinatorial way from a restricted set of items. In sentence comprehension, processing involves access to words organized into a syntactic structure: the meaning of the sentence derives from the meaning of the component words such as indicated by their association and sequential positioning [17]. Beyond the question *can a dog learn a word?* [18] is the question of whether dogs can integrate *several words* into a single, complex, directed performance.

Multiple-item, “sentence” processing have been obtained in nonhuman animals maintained in close contact and interaction with humans and subjected to training in linguistic skills through the use of human verbal or sign language, gestures or arbitrary signs such as lexigrams [19]: chimpanzees [20], bonobos, sea lions [21], bottlenosed dolphins [22], and african grey parrots [23] have been shown to correctly decode sentences composed, in variable combinations, of locations, actions, objects, objects features, recipients, etc., an indication of the existence of syntax-like processes.

Recent results on vocal production of primate species in the field suggest that combinatorial processes in communication are not restricted to animals trained by humans and that they may have a functional role in intraspecific, natural communication. For instance, Campbell monkeys combine basic loud calls into different sequences each associated with a highly specific context, such as travel, contact with conspecific groups, predators, etc., and each influencing in specific ways the behavior of other members of the group [24], [25].

We might expect dogs to show competence in receiving multiple-item verbal messages and to be able, like other linguistically trained animals, to translate the verbal components of requests (about locations, actions, objects, etc.) into integrated motor acts. This expectation is based on the growing evidence about dog's cognitive competence, especially in the social domain and on the special, domestication based sensitivity of dogs to human signals [26]. Dog owners actually report that their dogs obey multiple-word requests such as “let's have a walk” or “fetch the toy” which may however be responded to as individual signals: one of the component words may be selectively reacted to or the whole utterance may be taken as a single stimulus. Experimental uncoupling of sentence components is necessary to assess their differential role in behaviour.

A test in this direction was performed by Pilley and Reid [15], a paper that was published well after the moment our study was performed. In their Experiment 2, they presented the border collie Chaser with two-item requests in which three familiar action commands and three familiar objects were combined (none of the combinations had been previously used). Chaser's performance was correct in all 14 scheduled trials, a result which suggested that commands and nouns were endowed with independent meanings.

We provide here another evidence of a dog's ability to respond appropriately to two-item requests or sentences. Our experimental design, carried out with a single dog, Sofia, ensured that both items of each request, (an action term, *point* or *fetch*, and an object term, *key*, *ball*, *stick* or *bottle*, voiced in Portuguese) had to be taken into account as independent, yet connected components of the information provided to the dog, and that the dog's performance could not be attributed to the use of sentences as single items of information.

## Methods

### Ethics Statement

This study was conducted in accordance with the Ethical Principles in Animal Research adopted by the Ethical comitee for research with animals (CEPA), Psychology Institute, University of São Paulo, Brazil (no. 004/11-CEPA-IP).CEPA declaration regarding the study follows: *“The purpose of the experiment was to assess whether dogs may acquire, through multiple discriminative training, behaviors analogous to those that indicate syntactic comprehension in humans. A single dog, Sofia, two months of age at the start of the experiment, was submitted to training and test sessions, 2 or 3 times daily, during several months. The dog remained for the duration of the experiment at her owner's house, being handled the way pet dogs are normally handled. The complexity of the experimental tasks was increased gradually throughout the eight experimental phases (tasks involving the association of words with either acts or objects), and the dog was submitted to generalization tests (such as “unfamiliar experimenter”, “experimenter with dark glasses”, etc.). The experiment's theoretical interest justifies its execution. Procedures are original and do not involve any suffering to the animal. A single subject was used, under comfortable conditions at the lab, and under appropriate maintenance conditions at home. When the experiment was over, the dog remained at his owner's place. We consider that the study does not involve any infraction relatively to ethical principles in animal research and that it is an important contribution the study of symbolic communication in animals”.*

### Subject

Sofia, a female mongrel dog, 2 months old at the beginning of the experiment, was raised as a pet by a member of the research team and lived with him throughout the study. Training and testing procedures were carried out during 22 months so that Sofia was 2 years old when the experiment was over. She served in a simultaneously run experiment on arbitrary signals production [27].

### General Procedure

Training and testing sessions were conducted 2 to 3 times a day, 3 to 6 times a week in a dedicated room at the Institute of Psychology of the University of São Paulo, by a team of trainers.

Action terms *fetch* and *point* were selected because they would readily be installed in Sofia's repertoire as they can be in many dogs' behavior; object terms, *ball*, *key*, *stick*, *bottle* and *bear*, were selected from a set of sufficiently small and retrievable objects. *Ball*, *key*, *stick* and *bottle* were used during training; *bear* was introduced in phase 7 of the experiment to test for generalization of double-request performance.

Action and object terms were combined into two-item requests such as *ball fetch*, *key point*. Of the eight combinations of two action terms and four object terms (*ball fetch*, *ball point*, *key fetch*, *key point*, *bottle fetch*, *stick point*, *bottle point* and *stick fetch*), two (*bottle point* and *stick fetch*) were only used in a later phase of the experiment (phase 8), as a way of assessing Sofia's response to untrained two-item requests.

Treats, praise and petting – preceded by an audible “click” - were used to reward every correct performance. The clicker was pressed immediately after Sofia's response and served as a short-term feedback. Incorrect responses were followed by the word “no”: in this case the command was repeated until Sofia performed the correct response. Position of the objects throughout sessions was changed randomly according to a predefined schedule.

The experimental design included eight progressive phases, from the learning of verbal labels to the testing of comprehension of novel action-object requests. Very importantly, progression from one phase to the next and thus the number of sessions was not predetermined but depended upon the experimenters' evaluation of Sofia's level and stability of performance in ongoing tasks (i.e. when performance happened to keep stable, the phase was stopped).

### Training and Testing phases

#### Learning of single words (phase 1)

After a period of basic obedience training, four objects (*ball*, *key*, *bottle* and *stick*) were presented one at a time in a context of playful and informal interactions through which the learning of verbal labels was promoted. Sofia was rewarded for approaching an object the name of which was presented as part of a sentence (e.g. “what a beautiful *ball*”). Training of actions was done by requesting the dog to *point* or *fetch* familiar but unnamed objects (e.g. a plastic toothbrush, a rubber dog teether) and by rewarding correct performance.

#### Object and action training (phase 2)

In object training (109 sessions), restricted to two of the four objects set (*ball* and *key*), Sofia was rewarded for correctly approaching, when requested, a ball or a key, presented simultaneously. Objects were placed in transparent acrylic boxes, at each side of a wooden barrier, at one end of the experimental room. The experimenter stayed at the other end, at approximately 2.5 m from the objects. Sofia, initially facing the experimenter, had to turn back and walk towards the requested object. This was carried out in sessions of 20 requests (10 of each object term distributed randomly within session).

In action training (5 sessions), Sofia had to fetch (i.e. “bring back the object”) or point (“approach the object then stopping at it”) to the familiar but unnamed object presented singly in a transparent acrylic box. This was done in sessions of 30 requests, 15 of each action term randomly distributed within session. Since the objects were placed inside acrylic boxes, in order to fetch a given object, Sofia first knocked down the box thus reaching the object; to point at it, Sofia stood still by the box whilst pointing at the object with its nose.

#### Sequential object-action (phase 3)

In this stage (164 sessions, 20–40 trials per session), object and action requests were sequentially presented (*ball→fetch*, *ball→point*, *key→fetch*, *key→point*, *bottle→fetch* and *stick→point*). Each trial began with an object request. Following Sofia's approach to the correct object, an action request was then emitted. Whenever an incorrect object was selected, the trainer immediately blocked Sofia by saying “no”. Sofia then returned to the initial position and the object request was repeated (followed by the action request). Position of objects and requests were changed according to a predefined random schedule. The procedure was carried out by increasing progressively the number of objects involved: (1) two-object sessions (*ball*, *key*), (2) three-object sessions (ball, key, stick), (3) four-object sessions (ball, key, stick, bottle).

At the end of this phase, supplementary 2- and 3-object sessions (n = 19 sessions), with objects taken at random from the total set. This was added to the protocol because sessions with two objects in this phase only included *ball* and *key*; similarly, sessions with 3 objects only used *ball*, *key* and *stick*. Supplementary sessions with four objects were therefore unnecessary since the four-object sessions in this phase already included the total set of objects.

#### Simultaneous object-action (phase 4)

In this phase of the experiment (130 sessions, 20–40 trials per session), object and action items were combined into single requests (*ball fetch*, *ball point*, *key fetch*, *key point*, *bottle fetch* and *stick point*). While looking at the experimenter, Sofia was asked “ball point” (for instance) and was then released to respond. As in phase 3, 2-, 3- and 4-object sessions were scheduled, in this order.

#### Control tests (phase 5)

Further tests were run as controls for procedural and theoretical issues. To establish if Sofia's response to requests had been influenced by inadvertently produced cues and to test for performance generalization, 2-, 3- or 4- object sessions were run with simultaneous object-action requests, in the following conditions: (1) experimenter wearing sun-glasses, (2) experimenter with mouth covered by a cloth band, (3) research assistant absent from the room, (4) an unfamiliar person as experimenter, (5) testing in an unfamiliar room, (6) test objects scattered, distant from one another, (7) new objects of the same category (new balls, keys, etc.) offered. Tests were carried out in sessions of 20–40 requests, depending on the number of objects in the session. Only one type of session (i.e. 2-,3- or 4- object) was randomly selected for each condition.

#### Item reversal (phase 6)

To test for the possibility that performance was not actually guided by multi-item processing but was due to the learning of commands as single discriminative stimuli, we inverted the order of the sentence items, uttering action terms before objects ones. Sofia was thus required, in three 2-object sessions, to respond to simultaneous action-object sentences instead of object-action ones (*fetch ball*, *point ball*, *fetch key*, *point key*, *fetch bottle* and *point stick*); inverted commands were quite distinct acoustically from the original ones. In the first test session, the objects stick and key were used (total of 20 requests), in the second one, ball and bottle (total of 20 requests) and in the third session, ball and key (in a total of 20 requests). Inverted requests should not lead to correct performance, under a single stimulus hypothesis, as they are different in sound structure from original ones.

#### New object (phase 7)

Flexibility of processing was also tested by using a new object and its label as part of the request procedure. A teddy bear (*bear*) recently incorporated into Sofia's repertoire was used as the object in simultaneous object-action requests (*bear fetch* and *bear point*), in four 2-object sessions (with ball, key, stick, bottle and bear as alternatives). In the first test session, we used bear and stick as objects; in the second one, bear and bottle; in the third one, bear and ball and in the fourth one, bear and key. 20 requests were delivered per test session with 10 of them new (i.e. 5 “bear fetch” and 5 “bear point”).

#### New combination of items (phase 8)

As mentioned above, Sofia was never exposed, during two-item request training, to the combinations *stick fetch* and *bottle point*. Two test sessions with such new combinations were scheduled with 20 requests each. 10 novel combinations were delivered, 5 *stick fetch* during the first session and 5 *bottle point* during the second one. In both sessions, only stick and bottle, as objects, were used. In phase 8, correct responses were followed by a click, with no treats, petting or words as rewards.

### Analysis

Phase 1 was not analyzed as it constituted a informal phase of words introduction. In phases 2–4, total number of correct trials was recorded and analyzed. In phase 2, in which number of object trials differed from the number of action trials, we described Sofia's performance in the last five *object* sessions (150 trials) and the five *action* sessions (150 trials). In phases 3 and 4, to decrease a possible dependency of data obtained in successive trials, we took into account one in every five requests, in each session. Percentages of correct responses were compared to the chance levels using the Binomial Probability test (One-Sample Proportion Test).

In phases 6–7, besides taking the average scores in each test sessions, we described performance in the initial trials versus performance in the final trials, in order to evaluate possible learning effects within the sessions. We used Chi^2^ tests when comparing the performance in simultaneous and sequential requests.

## Results

### Object and action training (phase 2)

Sofia reached percentages of correct responses above chance levels for both object terms (i.e. 81,2% for *ball* and 84,4% for *key* considering the last 150 trials, being 75 *ball* and 75 *key*; One Sample Test, p<0.01). Sofia also reached percentages of correct responses above chance levels during the action training sessions, (i.e. 88% for *point* and 84% for *fetch*, considering 150 trials, being 75 *point* and 75 *fetch*; One Sample Test, p<0.01).

### Sequential object-action (phase 3)

Sofia's object choices (79,4%/n = 1090 trials, 67,9%/n = 1590 trials, 64,3%/n = 2160 trials) in 2, 3 and 4-object trials respectively) and action choices (93,6%/n = 1090 trials, 97,3%/n = 1590 trials, 98,3%/n = 2160 trials) in 2, 3 and 4-object trials respectively, were significantly above chance level (One Sample Test, p<0.01, all cases). Correct object and response choices in supplementary sessions, with objects selected at random, had similar values (object choices: 67,3%/n = 110 trials, 67,8%/n = 177 trials, in 2 and 3-object trials respectively; action choices: 98,2%/n = 110 trials, 100%/n = 177 trials, in 2 and 3-object trials respectively) and were also significantly different from chance levels (One Sample Test, p<0.01, all cases).

### Simultaneous object-action (phase 4)

**Figure 1:**
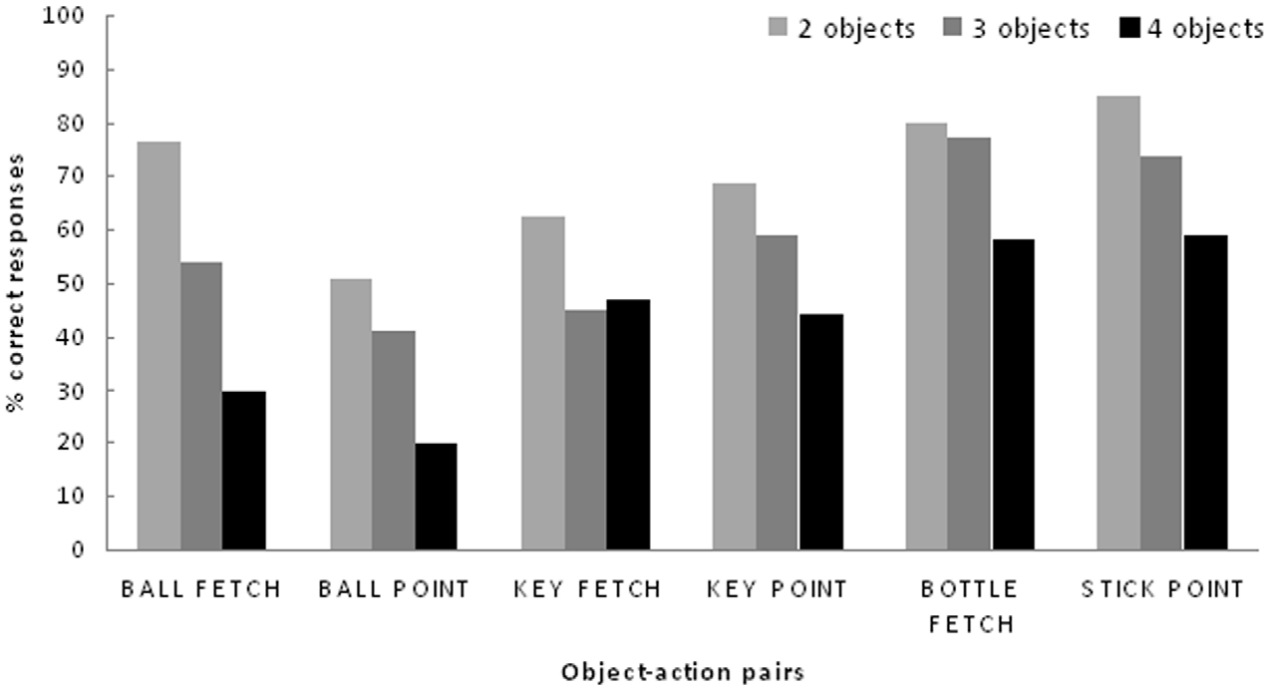
Percentage of correct responses for each of the object-action simultaneous requests (2-, 3- and 4- objects sessions). They were significantly above chance levels, One Sample Test, p<0.05, exception was *ball point* - p = 0.054).

Sofia responded correctly to the object term of requests on 85%/n = 1800 trails, 68.1%/n = 900 trials and 49.2%/n = 400 trials of times, and to the action term 90.6%/n = 1800 trials, 89.9%/n = 900 trials and 93.4%/n = 400 trials of times, in 2-, 3- and 4-object sessions respectively (One Sample Test, p<0.01, all cases). Most importantly, she performed above chance levels to object-action requests, e.g. she approached the right object and performed the right response towards it (72,5%/n = 1800 trials, 61,2%/n = 900 trials, 46%/n = 400 trials in 2-, 3- and 4-object sessions respectively, One Sample Test, p<0.05, all cases). Correct performance was significantly above chance levels in all but one object-action pairs (*ball fetch*, *key fetch*, *key point*, *bottle fetch and stick point*, Figure 1) in 2-, 3- and 4- object sessions.

Sofia's performance to simultaneous object-action requests was accurate from the very beginning, and it did not require training to reach significance. There was no significant difference in scores between the last 10 sessions of the sequential object-action phase and the first 10 sessions of the simultaneous object-action phase, in 2-object sessions (Chi^2^ = 2.099, p>0.05). A significant decrease in performance occurred, however, in 3-object (Chi^2^ = 7,356, p<0.01) and 4-object sessions (Chi^2^ = 35,014, p<0.001), when sequential requests were replaced by simultaneous ones.

Sofia's overall scores in 2-object sessions (Chi^2^ = 1,397, p>0.05) and 3-object sessions (Chi^2^ = 1,475, p>0.05) did not differ between sequential and simultaneous object-action phases. They were, however, lower in the simultaneous condition, in 4-object sessions (Chi^2^ = 40,759, p<0.001).

### Control tests (phase 5)

**Figure 2:**
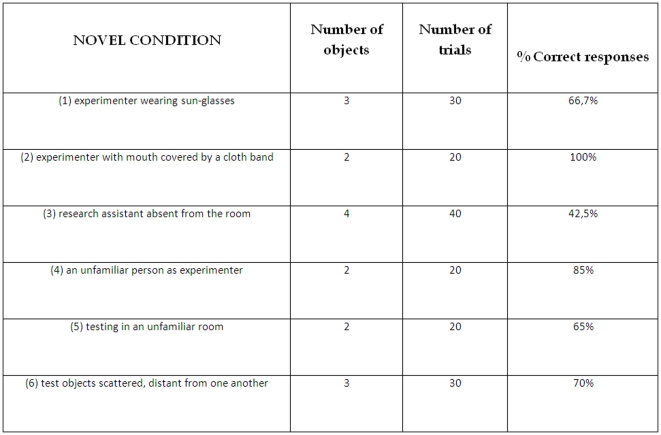
Percentage of correct responses for each of the novel condition test session (i.e. Control tests). All results were significantly above chance levels (One Sample Test, p<0.05).

Sofia performed significantly above chance in all cases (One Sample Test, p<0.01, all cases; see Figure 2). She also performed successfully in condition 7 (new objects condition), with 85% correct responses in the first session (new ball and new stick - One Sample Test, p<0.01), and 80% correct responses in the second session (new bottle and new key- One Sample Test, p<0.01), out of twenty trials in each of the sessions.

### Item reversal (phase 6)

Item reversal did not affect Sofia's performance. *Fetch ball* was as efficient a request as *ball fetch*. Overall performance level (42 correct responses out of 60 action-object requests) was not significantly different from performance obtained in the simultaneous object-action phase with 2-object sessions (Chi^2^ = 0.182, p>0.05). Sofia had 80% correct responses at the first session, 70% at the second session and 60% at the third session. Performance in the initial three trials was on average 73% correct responses whilst in the last three ones was 66% correct responses.

### New object (phase 7)

Correct performance (27 correct responses out of 40 object-action requests containing *bear*) was not significantly different from performance obtained in the simultaneous object-action phase with 2-object sessions (Chi^2^ = 0,489, p>0.05). Considering only the combinations containing the novel command *bear* she had 75% correct responses for *bear fetch* and 60% correct responses for *bear point*. Correct performance in the initial three trials was on average 60%; in the last trials was 70%.

### New combination of items (phase 8)

In the first session, Sofia had only 3 correct responses out of 10 *stick fetch* requests; in the second one only 3 correct responses out of 10 *bottle point* requests, a performance inferior to her previous overall performance in 2-object sessions.

## Discussion

Results of our study indicate that a dog may process independent items of verbal information provided in a single request and use them to organize sequentially her behavior. We showed that a complex utterance, made up by the combination of previously mastered terms, could control the dog's behavior such that a specified action would be performed on a specified object.

Previous work on language understanding by dogs has mostly centered on dogs' comprehension of verbal commands, taken as single communicative events and referring to whole situations and are not-syntactical in their form. In word recognition studies in which dogs are asked to retrieve one out of a set of objects, they, of course, both discriminate objects based on their labels and act in an appropriate way towards them (e.g. fetching), sometimes integrating information about object location [28]. Fetching or approach (“go to” commands) are however a constant feature of the task [12], [14], [28] and it is not possible, through the results of such studies, to evaluate the process by which action and object terms are separately taken into account and integrated into a successful performance. Such evaluation is obtained when, as in the present study, action and object are varied independently from one another.

It is here relevant, as in other experiments on complex communication, to rule out Clever Hans explanations. Sofia's performance could not be influenced by experimenter-produced cues about the object to be approached and the behavior to be performed towards it: turning back, after requests were voiced, she lost visual contact with the experimenter and had to rely exclusively on words.

Performance was not restricted to the specific training context and generalized to novel conditions: she was able to obey requests when non-semantic variables (visual access to the eyes or mouth of the experimenter [7]) were lacking; when requests were emitted by an unfamiliar person; when the spatial location of objects was changed, and when testing was done outside the laboratory. She generalized correct performance to objects in the same category, e.g., balls differing in size, shape and color and to a new object with a new label (bear). Such versatility suggests the existence of a capacity to extract and process relevant verbal features and a relative independence from contextual parameters.

Could Sofia's performance be accounted for by assuming that she learned, one by one, the correct responses to each object-action pairings of words in the whole set? Several aspects of our results make this assumption implausible: on one hand, the very quick and correct transition of performance from sequential to simultaneous requests which indicates that previously acquired responses tendencies were combined without or with little further training; on the other hand, and maybe more significantly, the maintenance of level of responding (at least with a small number of objects) when requests shifted from object-action to action-object. Under the hypothesis of separate learning of each combination, the reversal of items would be expected to decrease correct performance. Lack of improvement throughout test sessions also constitutes evidence against such hypothesis.

It is interesting to note that Sofia's performance did not reach a hundred per cent correct score in any session and also that there was a consistent decrease in performance, both in sequential and simultaneous object-action phases, as the number of objects used in a session increased (Figure 1). Both aspects may point to some constraint in Sofia's performance, maybe a difficulty in the discrimination of the spoken labels of objects; or a problem of memory which eventually grew higher when the number of objects presented increased.

Learning rate was higher with action terms than with object ones, a difference also obtained in an unpublished study in which eighteen dogs, submitted to training procedures similar to those used in the present experiment, were all shown to be able to acquire correct responses to action commands but failed, most of them, to master labels-objects associations [29]. This intriguing difference which deserves a more thorough examination, may relate to the history of dog breeding, during which many breeds were developed for herding, tracking, etc., cooperative tasks in which commands for action (not for object discrimination) prevail.

When presented with new combinations (*stick fetch* and *bottle point*) of previously mastered action and object terms, Sofia did not reach a successful level of responding. This result, based on a restricted number of trials, should be confirmed by new observations. It may (taking it at face value) derive from Sofia's previous exclusive training with *stick point* and *bottle fetch* requests, and might indicate the possible prevalence of a simpler, stimulus-bound way of reacting in cases of invariable non-combinatorial training conditions.

Sofia's processing of two-item sentences probably involves working memory processes, as occurs in human sentence comprehension [30]. In label training of dogs, responses happen in close temporal relationship to requests but do require the keeping of some information in memory (about what object is to be retrieved and where it is located). Performance under simultaneous requests depends on the storing of a more complex information (about object *and* action), the items of which must be put into use at appropriate stages of performance. Object-action requests are not obeyed by simply following the order in which terms were dispensed in the request sentence (object first, action second). One item of information is used first (*which is the appropriate object?*); the second one is used when near the object (*what is the appropriate action to be performed?*). It is thus conceivable that terms are stored in a parallel manner, independently of their order of reception, and are retrieved when certain environmental conditions are met. To retrieve information, Sofia uses her knowledge of the sequential structure of the task (an object must be approached before any action can be executed), and demonstrates understanding of the general principle that some actions require an object to be executed upon.

Sofia's prompt and successful performance for the new *bear fetch* and *bear point* requests gives a strong indication that, going beyond the learning of specific stimulus-response relationships, she was able to combine an action (selected among alternatives) to an object (selected among alternatives) even in the case of an object never before responded to with pointing or fetching. In an unpublished follow up experiment with Sofia, she was trained to choose either of two identical objects, placed at right or left in the experimental room and to perform either pointing or fetching towards it. Requests were thus action-action requests (*turn right or left* – *point or fetch*), not action-object ones. Sofia's performance in this task was highly successful and provided a confirmation of the dog's capacity to take into account and combine information items of a different nature.

Attention to the order of terms has been demonstrated in nonhuman species, in contexts in which the structural difference of sentences is relevant. Dolphins Akeakamai and Phoenix, for instance, when requested to *take the ball to the hoop*, pushed the ball until it got near the hoop and did the opposite, when requested to *take the hoop to the ball*. They were also able to learn different sequential grammars (S-V-O, Phoenix, O-V-S, Akeakamai) [31]. In Sofia's case, order of terms did not differentiate performance: *Fetch key* and *key fetch* were equivalent. Such equivalence may derive from training conditions which did not take order of items as a parameter. Further research may reveal to what extent dogs are able to discriminate the placement of terms in multiple-item requests.

Our results suggest that dogs share with “linguistic” animals [19]–[23] the capacity to encode in memory at least two heterogeneous items of information to be used in subsequent directed performance, a capacity which, although far from being “an infinite use of finite means” [32] as human grammars are, may have comparative relevance as a forerunner to syntactical functioning.
